# Usher Syndrome: New Insights into Classification, Genotype–Phenotype Correlation, and Management

**DOI:** 10.3390/genes16030332

**Published:** 2025-03-12

**Authors:** Fabiana D’Esposito, Giuseppe Gagliano, Caterina Gagliano, Antonino Maniaci, Alessandro Avitabile, Rosa Giglio, Michele Reibaldi, Maria Francesca Cordeiro, Marco Zeppieri

**Affiliations:** 1 Imperial College Ophthalmic Research Group (ICORG) Unit, Imperial College, 153-173 Marylebone Rd., London NW1 5QH, UK; f.desposito@imperial.ac.uk (F.D.);; 2 Department of Neurosciences, Reproductive Sciences and Dentistry, University of Naples Federico II, Via Pansini 5, 80131 Napoli, Italy; 3 Eye Clinic Catania University San Marco Hospital, Viale Carlo Azeglio Ciampi, 95121 Catania, Italy; 4 Department of Medicine and Surgery, University of Enna “Kore”, Piazza dell’Università, 94100 Enna, Italy; 5 Mediterranean Foundation “G.B. Morgagni”, Via Sant’Eupio, 95100 Catania, Italy; 6 Department of Medicine, Surgery and Health Sciences, University of Trieste, Via Farneto 3, 34100 Trieste, Italy; 7 Department of Surgical Sciences, University of Turin, Corso Bramante 88, 10126 Turin, Italy; 8 Department of Ophthalmology, “City of Health and Science” Hospital, 10126 Turin, Italy; 9 Department of Ophthalmology, University Hospital of Udine, p.le S. Maria della Misericordia 15, 33100 Udine, Italy

**Keywords:** Usher syndrome, syndromic hearing loss, rod-cone dystrophy, genetic variants, genotype–phenotype correlations, next-generation sequencing, personalized treatment

## Abstract

Background: Usher syndrome (USH), the most common cause of combined deaf-blindness, is a genetically and phenotypically heterogeneous disorder characterized by congenital hearing impairment and progressive vision loss due to rod-cone dystrophy. Although the original classification in three subtypes (USH I, USH II, and USH III) is still valid, recent findings have changed and widened perspectives in its classification, genotype–phenotype correlations, and management strategies: Objective: This study aims to provide new insights into the classification of Usher syndrome, explore the genotype-phenotype correlations, and review current and emerging management strategies. Methods: A comprehensive literature review has been conducted, incorporating data from clinical studies, genetic databases, and patient registries. Results: Recent studies have led to the identification of several novel pathogenic variants in the USH genes, leading to refined subclassifications of Usher syndrome. Interactions between different genes being part of the network of this ciliopathy have been investigated and new mechanisms unveiled. Significant correlations were found between certain genotypes and the presentation of both auditory and visual phenotypes. For instance, pathogenic variants in the *MYO7A* gene (USH1B) were generally associated with more severe hearing impairment and earlier onset of retinal dystrophy, if compared to other USH genes-related forms. Other genes, such as *USH1G*, traditionally considered as causing a specific subtype, can display phenotypic heterogeneity in some patients. Conclusions: This review provides insights into a better understanding of Usher syndrome that considers recent findings regarding its genetic causes and clinical features. Precise genotype–phenotype correlations can lead to better genetic counselling, more precise characterization of the natural history of the condition, and a personalized and effective management approach. Recent progress has been made in research into gene-specific therapies that appear promising for improving the quality of life for individuals affected by Usher syndrome.

## 1. Introduction

Usher syndrome (USH) is a genetically determined condition with an estimated prevalence of 1/10.000, resulting in congenital or early-onset sensorineural hearing loss and progressive retinal degeneration in the form of rod-cone dystrophy; variable vestibular impairment may be associated in the type I form [1].

USH is inherited in an autosomal recessive manner and is caused by pathogenic variants in at least 16 genes [2] involved in the function of inner ear hair cells and in the maintenance of photoreceptors cells in the retina. The underlying pathophysiology reflects a ciliopathy, where dysfunctional proteins alter transduction in cochlear hair cells and defective morphogenesis in photoreceptors [3]. Genes involved in USH pathogenesis encode a variety of proteins, including cadherins, protocadherins, myosins, scaffolding proteins, and transmembrane receptors, which collectively form a network essential for proper ciliary function.

Classically, USH is divided into three subtypes (USH I, USH II, and USH III), based on the severity and onset of hearing loss and the presence of vestibular dysfunction, and an additional USH IV form has been recently described, distinguished by late-onset hearing loss and retinal dystrophy, while maintaining vestibular function [4]. Recent studies suggest that USH is not simply a static triad of subtypes, but rather a heterogeneous spectrum of conditions resulting from a growing number of pathogenic variants within an expanding array of genes [5].

## 2. Materials and Methods

A systematic literature search was performed for this narrative review in January 2025, utilizing PubMed (https://pubmed.ncbi.nlm.nih.gov), Medline (https://www.nlm.nih.gov/medline), the Cochrane Library (https://cochranelibrary.com), and ClinicalTrials.gov (https://clinicaltrials.gov). Search terms encompassed ‘Usher syndrome’, ‘retinitis pigmentosa’, ‘hearing loss’, ‘retinal degeneration’, ‘gene therapy’. Boolean operators(‘AND’, ‘OR’) were employed to enhance the specificity of the results.

Following a preliminary evaluation of titles and abstracts, 72 items were eliminated. The principal grounds for exclusion comprised studies concentrating on non-genetic facets of Usher syndrome (n = 22), review articles lacking novel data (n = 15), case reports with restricted generalizability (n = 10), conference abstracts devoid of comprehensive peer-reviewed data (n = 13), and studies with inadequate methodological specifics (n = 12). The final 29 publications were chosen for their pertinence to the genetic foundation, genotype–phenotype relationships, and therapeutic progress associated with Usher syndrome. Moreover, additional references were discovered by focused searches for certain genes and disease pathways, guaranteeing a thorough and current assessment.

Updated information about different genes and genotype–phenotype correlations has been verified on RetNet (https://retnet.org/) and OMIM (https://www.omim.org/).

## 3. Results

The phenotypic and genetic characteristics displayed by the different Usher syndrome subtypes follow.

### 3.1. Usher I Syndrome

#### 3.1.1. Phenotype

Usher I Syndrome (USH I) is the most severe form of Usher syndrome, a rare autosomal recessive disorder characterized by the co-occurrence of sensorineural hearing loss (SNHL) and visual impairment. It accounts for approximately 25–44% of all cases of Usher syndrome, making it the most prevalent form of this condition [6].

One of the hallmark characteristics of USH I is profound, congenital, bilateral sensorineural hearing loss, which is present from birth. The hearing loss is usually profound and remains stable throughout the individual’s life. This form of hearing loss is caused by dysfunction of cochlear hair cells. Since the auditory impairment is congenital and permanent, children with USH I often require early intervention, including cochlear implants or hearing aids, to assist in communication and auditory development. This profound hearing loss is one of the most significant challenges faced by individuals with USHI and leads to delays in language development if not addressed early [7].

Another distinctive feature of USH I is vestibular areflexia, which refers to the absence of normal responses in the vestibular system that are responsible for maintaining balance and spatial orientation. Vestibular areflexia leads to severe motor developmental delays in affected children, who often do not begin to walk until after 18 months of age [6]. This delay is due to the inability to sense balance and spatial positioning, resulting from the absence of functional vestibular reflexes [8]. As a result, children with USH I may display uncoordinated movements and a higher risk of falls. To compensate for this vestibular dysfunction, affected children frequently rely on their vision for spatial orientation and balance [9]. However, as the disease progresses and retinitis pigmentosa (RP) begins to affect vision, this compensatory mechanism becomes less effective, and motor function continues to deteriorate [10].

The third major component of USH I is the early onset of retinitis pigmentosa (RP), a degenerative rod-cone retinal disease that leads to progressive vision loss. The onset of RP in USH I typically occurs in early childhood and follows a distinct progression. Night blindness (nyctalopia) is the first visual symptom, typically presenting in the first decade. This impairment is due to the degeneration of rod photoreceptors, which are responsible for vision in low-light conditions. Loss of rod function impairs the ability to see in dim light, leading to significant difficulties in navigating at night. As RP progresses, the degeneration of the rods extends from the retinal periphery towards the center, causing a gradual reduction of the visual field, also known as “tunnel vision” [11].

The visual symptoms of RP in USH I typically progress in a predictable sequence: first, night blindness, followed by peripheral vision loss (tunnel vision), and, eventually, central vision loss. The degeneration of rods and cones is gradual but irreversible, and while the speed of progression can vary among patients, the ultimate outcome is usually complete blindness by the third decade of life [12]. The rate of decline in visual acuity and visual field loss can be influenced by genetic factors, but it remains progressively debilitating in all cases [3].

#### 3.1.2. USH I Underlying Genes

The genetic basis of USH I has been well characterized, with several key genes identified as contributing to the pathogenesis of this condition (Table 1) [13].

One of the first loci identified as causative of Usher syndrome was USH1A, localized at the cromosome 14q32 region, although the specific gene for USH1A remains unidentified [14].

*MYO7A* (Myosin VIIA) is the most frequently underlying gene in USH I, accounting for approximately 50–60% of cases: These cases are identified as an USH I subtype named as the locus, USH1B. This gene encodes myosin VIIA, a motor protein that is essential for the function of hair cells in the inner ear and photoreceptors in the retina [15]. Myosin VIIA is involved in the transport of essential proteins and in the maintenance of cellular structures, including the stereocilia of hair cells, which are crucial for auditory transduction. Pathogenic variants in *MYO7A* impair the function of these cells, leading to the profound sensorineural hearing loss seen in USH I. Additionally, *MYO7A* impairment affects photoreceptors, leading to retinitis pigmentosa and progressive visual loss. *MYO7A*-related USH I typically leads to early onset of night blindness and a rapid progression of visual field constriction, eventually leading to severe impairment or loss of visual acuity [16]. *MYO7A* can also cause isolated hearing loss, without signs of RP [17].

Pathogenic variants in *CDH23* (Cadherin 23) cause approximately 10–15% of type I cases and they identify USH1D subtype. *CDH23* encodes a protein of the cadherin family, which plays a critical role in maintaining the stability of cell junctions in both the inner ear and the retina. In the cochlea, CDH23 is involved in the formation of stereocilia in hair cells, while in the retina, it contributes to retinal cell adhesion. Defects in *CDH23* disrupt these processes, resulting in sensorineural hearing loss and visual impairment. Patients with *CDH23* pathogenic variants tend to have a slightly less severe phenotype in terms of both hearing loss and vision impairment compared to *MYO7A*-related forms [18].

USH1F subtype is caused by pathogenic variants in *PCDH15* gene (Protocadherin 15), implicated in approximately 10% of USH I cases. It encodes a protocadherin protein, which facilitates cell adhesion in both the inner ear and the retina. The role of PCDH15 in the integrity of hair cells and photoreceptors is essential for normal auditory and visual function. Pathogenic variants in this gene result in a classic USH1 phenotype. The severity of RP in *PCDH15*-related USH1 may vary, but like other forms of USH I, it leads to eventual total loss of visual function [19].

*USH1C* pathogenic variants are responsible for approximately 3–5% of USH I cases. *USH1C* encodes a scaffold protein involved in intracellular trafficking and WNT signaling [20]. These proteins play a key role in ensuring the stability and function of stereocilia in the inner ear and the retinal photoreceptors. Pathogenic variants in *USH1C* result in early-onset sensorineural hearing loss, as well as the gradual development of rod-cone dystrophy. The phenotype associated with *USH1C* may include earlier onset of night blindness and a more rapid progression of RP, although the rate of visual decline varies among affected individuals.

Pathogenic variants in *SANS*, also defined as *USH1G*, are rare, accounting for less than 5% of all USH I cases [21]. The SANS protein is involved in the stability of sensory cells, particularly in the inner ear. Disruption of SANS function results in defective hair cells in the cochlea and to impairment in the photoreceptors function. The progression of both hearing and vision impairment in SANS-related USH I is typically consistent with other forms of USH I, leading to total blindness in early adulthood [22].

*CIB2* is a widely expressed gene, particularly in the retina (RPE and photoreceptors) and in the inner ear, with a role in the Usher interactome. It has been reported as a possible cause of USH1 [23]. This role has been debated, and in 2018, Booth et al. [24] described a cohort of patients affected by non-syndromic hearing loss (NSHL) related to pathogenic variants in *CIB2*, without any evidence of retinal dystrophy.

In addition to the well-characterized genes underlying Usher syndrome type I, two more loci, USH1E and USH1H, have been identified; however, their causal genes remain uncharacterized. USH1E locus (OMIM #602097) is located on chromosome 21q21, and USH1H (OMIM #612632) maps on chromosome 15q22-23. These loci were identified in families exhibiting an Usher syndrome type I phenotype, including congenital profound hearing loss, vestibular dysfunction, and early-onset retinal dystrophy. Despite great efforts, the precise genes accountable for these subtypes remain unidentified to date [25,26].

#### 3.1.3. Genotype–Phenotype Correlations

While all forms of USH I are defined by the combination of profound hearing loss, vestibular dysfunction, and retinitis pigmentosa, the severity and progression of these features can differ among individuals depending on the underlying genetic defect.

*MYO7A* pathogenic variants are associated with the most severe phenotypes, characterized by profound hearing loss at birth, complete vestibular areflexia, and rapid progression of retinitis pigmentosa, although usually the temporal sector of visual field can remain partially preserved until later stages [12]. Most individuals with *MYO7A*-related USH1 experience total blindness by their 20s and have severe delays in motor development due to vestibular dysfunction combined with visual impairment. Early intervention with cochlear implants or hearing aids is essential to improve communication and development, but visual decline remains progressive.

*CDH23* and *PCDH15* pathogenic variants tend to result in a milder phenotype compared to *MYO7A*. Individuals with alterations in these genes often retain some functional vision into their 30s or even 40s, with slower progression of retinitis pigmentosa. Hearing loss is also profound, but vestibular dysfunction can be less severe, leading to milder motor delays compared to *MYO7A* mutations. *CDH23*-related USH1 may also have a later onset of RP, with less severe visual impairment in the early years of life.

USH1G can display a USH2-like phenotype, with patients lacking vestibular symptoms, and with a milder phenotype compared to the classical USH I [27].

### 3.2. Usher II Syndrome

Usher syndrome type II (USH II) primarily presents with moderate-to-severe sensorineural hearing loss from birth, a progressive form of retinitis pigmentosa, and a largely unaffected vestibular system. In contrast to Usher syndrome type I, where profound hearing loss and balance impairment are common, USH II is characterized by residual hearing that responds well to hearing aids or cochlear implants and a normal vestibular function [28]. However, the onset and progression of retinal degeneration can vary widely, underscoring the complexity of this disorder.

#### 3.2.1. Phenotype

Patients with USH II typically show hearing impairment that is noticeable from birth or early childhood. The degree of loss is usually moderate to severe, though variability is common even within the same family, and in some patients, the hearing impairment can even remain undiagnosed. Standard audiometric tests often reveal a pattern where higher frequencies are more affected than lower ones. This residual hearing sets USHII apart from the severe congenital deafness seen in USH I, allowing many USH II patients to achieve good benefit from hearing devices [29]. 

The retinal degeneration seen in USH II is a form of retinitis pigmentosa, primarily involving the rod photoreceptors. Early symptoms include difficulties seeing in dim light (nyctalopia), with patients often noticing these changes during adolescence or early adulthood. As the disease advances, cone photoreceptors are also affected, leading to the gradual loss of central vision. The progression rate is not uniform, with some individuals maintaining central vision for many years, while others can experience a more rapid deterioration. Typical fundoscopic findings include bone spicule pigment deposits, narrowed retinal vessels, and a pale optic disc [30].

A defining aspect of USH II is the preservation of vestibular function. Unlike in USH I, where severe balance dysfunction is a hallmark, patients with USH II generally exhibit normal balance. This intact vestibular function is evident in normal motor development milestones, such as walking at the expected age [1].

#### 3.2.2. Underlying Genes

USH II is genetically heterogeneous, with several key genes related to the phenotype. The primary genes implicated are *USH2A*, *ADGRV1* (also known as *GPR98*), and *WHRN* (Table 2) [31]. The proteins encoded by these genes play essential roles in both the inner ear—particularly in the structure and function of hair cell stereocilia—and the retina, where they support photoreceptor cell stability [32].

The *USH2A* gene, located on chromosome 1q41, is the most frequently mutated gene in USH II. It encodes usherin, a large transmembrane protein that contributes to cell adhesion, extracellular matrix interactions, and structural support within photoreceptors and inner ear hair cells. Given its extensive structure (over 70 exons), *USH2A* is susceptible to a broad spectrum of pathogenic variants, including nonsense, frameshift, and missense changes. Certain regions, like exon 13 in some populations, tend to be mutation hotspots, which may explain a consistent prevalence of pathogenic variants related to this particular genetic region [33]. Numerous studies in large cohorts of ethnically different populations have identified pathogenic compound heterozygous or homozygous variants in *USH2A* as the most frequent cause of isolated recessive RP, without any sign of hearing impairment [34,35,36]. Phenotypic heterogeneity seems to reflect an allelic hierarchy mechanism [37].

Located on chromosome 5q14.3, *ADGRV1* encodes for an adhesion G protein-coupled receptor known as VLGR1. This receptor contains multiple calcium-binding EGF-like domains and large extracellular adhesive regions that are critical for maintaining the integrity of cochlear hair cells and retinal photoreceptors. Pathogenic variants in *ADGRV1* can lead to disorganized stereocilia bundles, impairing the mechanotransduction process and resulting in sensorineural hearing loss. Although disease-causing variants in *ADGRV1* are less common than those in *USH2A*, the clinical presentation remains largely similar [38], although perimacular areas of atrophy and relatively retained macular structure in later adulthood are more often seen in *ADGRV1*-related, compared to *USH2A*-related, USH forms [39].

The *WHRN* gene, on chromosome 9q32, produces whirlin—a protein with several PDZ domains that act as scaffolds within the hair cell stereocilia. Whirlin is vital for the normal elongation and upkeep of these structures in the inner ear, and it has a comparable role in the retina. Defects in *WHRN* disrupt key protein interactions, leading to abnormalities in both hair cells and photoreceptors that manifest as the USH II phenotype. Although less frequently responsible, *WHRN* remains a crucial gene to consider, especially when *USH2A* pathogenic variants are not detected [40].

The overall heterogeneity of USH II suggests that additional genetic factors—including modifier genes and potential oligogenic effects—might influence disease severity and progression. Some researchers have proposed that interactions among variants in multiple genes contribute to the wide clinical spectrum observed [41].

**Table 2:** Usher II syndrome subtypes, related genes [17], and percentage of patients genetically characterized [31,42].

Locus	Gene	Percentage
USH2A	*USH2A*	57–79%
USH2C	*ADGRV1*	6.6–19%
USH2D	*WHRN*	0–9.5%

#### 3.2.3. Genotype–Phenotype Correlations

Establishing direct correlations between specific genetic variants and clinical outcomes in USH II remains challenging. The overlapping clinical features, diverse mutation spectrum, and influence of external factors all contribute to this complexity [43].

Generally, pathogenic variants in *USH2A* are linked to the classic USH II profile: moderate-to-severe hearing loss with RP emerging during adolescence or early adulthood. Some studies suggest that truncating mutations (such as nonsense or frameshift changes) may be tied to an earlier and more severe loss of visual function, while missense mutations might be associated with a milder, slower progression. Nonetheless, significant variability, even within families, has been documented [44].

Individuals with *ADGRV1*-related USH II often present with a clinical picture that closely resembles *USH2A*-associated cases in terms of both hearing loss and retinal degeneration. Although there are occasional reports of an earlier decline in visual acuity, these findings are not consistently observed across all studies. Overall, the degree of hearing impairment remains moderate to severe with little further deterioration over time [31].

Cases attributed to *WHRN* defects (sometimes labeled USH2D) tend to follow the typical USH II presentation. However, some patients may show subtle differences, such as variations in the high-frequency hearing loss or differences in the onset of RP symptoms. Disruptions in whirlin’s structural role may influence the pace at which photoreceptor degeneration occurs [45].

### 3.3. Usher III Syndrome

#### 3.3.1. Phenotype

Usher syndrome type III (USH III) is the least common form of Usher syndrome, comprising less than 5% of all cases worldwide. It is characterized by a progressive combination of sensorineural hearing loss, retinitis pigmentosa (RP), and, in some cases, vestibular dysfunction. Unlike USH I and USH II, USH III displays a highly heterogeneous phenotype, with considerable inter- and intra-familial variability in clinical manifestations. The disease follows an autosomal recessive inheritance pattern [46]. The clinical course of USH3 is unpredictable, as symptoms can present at different ages and progress at varying rates. Some patients may experience early-onset rapid deterioration, while others retain partial hearing and vision well into adulthood. The presence of genetic modifiers and environmental factors is believed to contribute to this variability, making personalized treatment strategies particularly important for disease management [47].

Patients with USH III typically present with mild-to-moderate sensorineural hearing loss during childhood or adolescence. This impairment is progressive, leading to profound deafness in adulthood if untreated. The rate of auditory decline varies among individuals: Some experience rapid deterioration within a few years, while others retain residual hearing for extended periods [47]. Cochlear implantation has been demonstrated to significantly improve auditory perception and communication outcomes, emphasizing the importance of early intervention [48,49]. Visual impairment in USH III predominantly manifests as RP, a progressive degeneration of retinal photoreceptors. Initial symptoms include nyctalopia and gradual peripheral vision loss, progressing to tunnel vision and, in severe cases, complete loss of central vision. The age of onset and rate of progression are highly variable, influenced by genetic modifiers and environmental factors such as oxidative stress and exposure to high-intensity light. Studies indicate that oxidative stress accelerates photoreceptor degeneration, suggesting the importance of antioxidant-based protective strategies such as dietary supplementation and light protection measures [50]. Vestibular involvement in USH III is inconsistent. Some patients exhibit balance difficulties and delayed motor milestones, whereas others retain normal vestibular function. Histopathological studies have identified significant microstructural alterations in the inner ear, indicating that degeneration extends beyond the auditory system to include vestibular structures. These deficits contribute to motor instability, which worsens as hearing and vision deteriorate. Karjalainen et al. investigated vestibular function in patients with USH III, showing that some individuals present with abnormal electronystagmography (ENG) findings, even before evident balance issues emerge. This suggests that vestibular dysfunction may be an early indicator of disease progression and could serve as a diagnostic marker [51].

#### 3.3.2. Underlying Genes

USH III is primarily associated with mutations in the *CLRN1* (clarin-1) gene, located on chromosome 3q21–q25. Clarin-1 is a transmembrane protein crucial for the structural integrity and function of cochlear hair cells and retinal photoreceptors. It plays an essential role in synaptic function and mechanotransduction, fundamental processes for auditory and visual perception [52].

*HARS* is an additional gene that has been related to an USH III phenotype, in a more complex syndromic form, also including motor development delay, truncal ataxia, and gait abnormalities [53].

#### 3.3.3. Genotype–Phenotype Correlations

Variations in *CLRN1* directly influence the progression and severity of hearing loss, retinitis pigmentosa, and vestibular dysfunction. Patients harboring the c.144T>G variant experience a severe phenotype, often presenting with rapid auditory decline and early-onset RP. The structural consequences of this mutation lead to significant dysfunction in cochlear hair cells and photoreceptors, accelerating the progression of the disease. In contrast, c.528T>G variants generally result in a milder phenotype, with a delayed onset and slower sensory degeneration, potentially due to residual clarin-1 functionality [10].

### 3.4. Usher IV Syndrome

#### 3.4.1. Phenotype

Usher syndrome type IV (USH IV) is an ultra-rare form of Usher syndrome, distinct from the classical USH I, USH II, and USH III subtypes. Its defining characteristics include late-onset sensorineural hearing loss (SNHL) and retinitis pigmentosa (RP) with a delayed onset compared to other Usher subtypes [47,54]. 

Unlike USH I, where the deafness is congenital and profound, and USH II, displaying a congenital and moderate hearing loss, USH I IV presents with progressive hearing loss that begins in adulthood, typically in the fourth or fifth decade of life. Retinitis pigmentosa in USH4 develops later than in other Usher types, usually appearing around the fifth decade. The retinal degeneration follows a rod-cone dystrophy pattern, with initial peripheral vision loss progressing towards the macula. USH4 does not significantly impact balance: Patients generally maintain normal vestibular function throughout life.

Fundoscopic examination typically reveals ring-shaped atrophy along the vascular arcades, a distinctive feature in USH4. Optical coherence tomography (OCT) often shows perifoveal outer retinal layer loss, and full-field electroretinography (ERG) exhibits reduced rod and cone responses [4].

#### 3.4.2. Genotype

USH IV has been genetically linked to pathogenic variants in the *ARSG* (Arylsulfatase G) gene, located on chromosome 17q24. ARSG encodes a lysosomal sulfatase enzyme, which is involved in glycosaminoglycan degradation. Like other Usher types, USH IV follows an autosomal recessive inheritance mode [4].

#### 3.4.3. Genotype–Phenotype Correlation

Unlike USH I-III, where genotype–phenotype correlations are well established, USH IV remains poorly characterized due to its rarity. However, some trends have emerged: Loss-of-function *ARSG* pathogenic variants (e.g., nonsense or frameshift variants) correlate with earlier onset and faster progression of RP. Milder missense variants may be associated with a later onset of retinal degeneration. One unique aspect of USH IV is the absence of neurological symptoms typically associated with lysosomal storage disorders, despite ARSG’s role in lysosomal metabolism [55,56].

### 3.5. Rare Genes

Several other genes have been related to Usher Syndrome. The better characterized are briefly described:

*ABHD12*: Pathogenic variants in *ABHD12* are known to cause a complex syndrome characterized by polyneuropathy, hearing loss, ataxia, retinitis pigmentosa, and cataract (PHARC). An *ABHD12* pathogenic variant was identified in a Lebanese family with USH3-like findings and cataracts [57].

*CEP250*: A homozygous nonsense variant in *CEP250* accompanied by a heterozygous or homozygous nonsense variant in *PCARE* was found to be related to an atypical Usher syndrome in a consanguineous family [58]. The *PCARE* variant seems to increase severity in an additive mode. It is not clear if *CEP250* variants alone may or may not be sufficient to cause Usher syndrome [59].

*CEP78*: Homozygous and compound heterozygous *CEP78* variants have been found to cause cone-rod dystrophy in some families. Two consanguineous Chinese families with homozygous *CEP78* variants were reported to have atypical Usher syndrome with adolescent or late-onset neurosensory hearing loss [60].

*ESPN*: A homozygous deletion in the *ESPN* gene was found in a large, extended, consanguineous Pakistani family with prelingual hearing loss, vestibular dysfunction, and retinal dystrophy consistent with a diagnosis of Usher syndrome type I [61].

*AGBL5*: recently described as underlying syndromic cases of combined retinitis pigmentosa and hearing loss [62].

### 3.6. Principal USH Genes—Protein Localization and Function

The proteins expressed by genes related to Usher syndrome are crucial for the functioning of sensory cells in the inner ear and retina. MYO7A is defined as an unconventional myosin: an actin-based motor protein, essential for transport in cochlear hair cells and photoreceptor cells, maintaining the integrity of stereocilia and the photoreceptor ciliary structure [63]. USH1C, harmonin, is a PDZ-domain scaffold protein [64].

PCDH15 and CDH23 are adhesion proteins that play a crucial role in the creation of tip linkages in hair cells, essential for mechanoelectrical transmission, and seem to have a role in the regulation of stereocilia lengthening [65]. USH1G/SANS plays a role in the development and maintenance of the auditory and visual systems and functions in the cohesion of hair bundles formed by inner-ear sensory cells [27]. CIB2 protein is part of the USH interactome and is essential for the function and proper development of hair cells and retinal photoreceptor cells.

Likewise, USH2A, which encodes usherin, is expressed in photoreceptor cells and cochlear hair cells, playing a role in structural integrity and cellular adhesion. ADGRV1 and CLRN1 participate in the elongation of stereocilia and the maintenance of photoreceptor homeostasis. WHRN is thought to function in the organization and stabilization of sterocilia elongation and actin cystoskeletal assembly [66].

In photoreceptors, while MYO7A, USH1C, USH1G, CDH23, and PCDH15 are expressed at the base of outer segments, USH2A and ADGVR1 are expressed in the ridge complex [5].

### 3.7. Comparative Phenotypic Overview

Usher syndrome is clinically categorized into four subtypes, according to the severity of hearing loss and retinal degeneration, and the presence of vestibular impairment. Usher syndrome type I is characterized by severe congenital sensorineural hearing loss, vestibular areflexia resulting in delayed motor development, and early-onset retinitis pigmentosa, usually presenting with night blindness and visual field reduction within the first decade of life. Patients affected by Usher syndrome type II display moderate-to-severe congenital hearing impairment, normal vestibular function, and a delayed onset of retinitis pigmentosa, typically manifesting during the second decade of life. Usher syndrome type III is characterized by gradual hearing loss that usually manifests in childhood or adolescence, varied vestibular dysfunction, and retinitis pigmentosa with variable onset and progression. Finally, Usher syndrome type IV, the most uncommon variant, is characterized by adult-onset hearing impairment, late-onset retinitis pigmentosa, and normal vestibular function. This phenotypic range highlights the clinical variability of Usher syndrome (Table 3) [54].

### 3.8. Therapeutic Approaches

The development of therapeutic strategies for Usher syndrome has significantly advanced over the past decades with a focus on gene therapy, particularly for *MYO7A*-related Usher syndrome (USH1B).

Lopes and Williams (2015) [67] reviewed the challenges and progress in gene therapy for USH1B, highlighting the size limitations of *MYO7A*, which exceeds the packaging capacity of traditional adeno-associated virus (AAV) vectors. To overcome this, researchers have explored lentiviral vectors with larger capacity and dual-AAV vector systems, which split the *MYO7A* gene into two parts that recombine upon delivery. Preclinical studies demonstrated that these approaches successfully restored retinal pigment epithelium (RPE) function and preserved photoreceptor integrity in animal models [68]. These findings underscore the potential of gene therapy to halt or slow down the progression of retinal degeneration in USH1B patients, making it a leading candidate for clinical translation [15,67].

Girach et al. [69] reviewed the emerging role of RNA-based therapies in treating inherited retinal diseases, including Usher syndrome. Their study highlights how antisense oligonucleotides (ASOs) and RNA editing strategies are becoming key therapeutic options for retinal degeneration, offering alternatives to traditional gene replacement approaches. ASOs are short synthetic RNA molecules designed to modulate pre-mRNA splicing, allowing cells to bypass premature stop codons or restore correct protein expression. The article outlines several RNA-targeted approaches currently being explored for Usher syndrome, with a focus on *USH2A*: a clinical trial in patients with pathogenic variants in *USH2A* gene exon 13 (NCT05158296) terminated in 2024 due to business-related decisions.

Fuster-García et al. [70] investigated CRISPR/Cas9-based gene editing as a potential therapy for *USH2A* mutations. The study targeted exon 13 mutations, which frequently lead to retinitis pigmentosa (RP). Using in vitro models, researchers successfully designed and validated CRISPR/Cas9 guide RNAs to precisely edit *USH2A* mutations. Their findings demonstrated efficient and specific gene correction, reducing the production of defective usherin protein. This study provides a foundation for future gene-editing therapies in *USH2A*-associated RP, though further preclinical and clinical validation is required to ensure safety and efficacy before human application.

Others therapeutic approaches include translational read-through therapy, a gene-based approach that suppresses protein truncation by allowing the translation machinery to bypass premature stop codons: This therapy has shown effectiveness in restoring USH1C and USH2A protein levels in preclinical studies. For advanced retinitis pigmentosa (RP), alternative treatments include cell replacement therapy, using embryonic stem cells and optogenetics, which introduces light-sensitive proteins into non-functional retinal cells [71].

## 4. Discussion

Usher syndrome represents a broadly investigated condition in the scientific world. Its severity, coupled with its considerable prevalence, has made it an attractive disease for scientists and investors in research fields through the years.

As for most recessive diseases, the geographical distribution of the different genes-related forms is influenced by the presence of a high level of consanguinity in certain areas, where some pathogenic variants are specifically present and significantly prevalent [72].

Particularly, being the principal cause of deaf-blindness [30], it has been widely investigated by both ophthalmologists and audiologists, and numerous studies can be found in the literature, describing the most diverse populations. In addition, being mainly considered a ciliopathy, it is the focus of numerous scientists, who are interested in the elucidation of complex mechanisms underlying this category of conditions [73].

Progress in understanding molecular mechanisms, and towards the appointment of therapeutic strategies, has been facilitated by the availability of numerous animal models. To date, there is an increasing number of trials that are being undertaken to investigate therapeutic strategies [74,75].

While researchers are focusing on the possibility of finding a therapy for Usher syndrome, a considerable number of patients still remain without a clinical and/or a molecular diagnosis. Although scientists and clinical professionals in tertiary care specialized clinics are well aware of the importance of a proper characterization and genotype–phenotype correlation, we still encounter patients for whom the audiological and visual impairments have not been put in relation, or who have been only clinically diagnosed with only one of the phenotypes, especially in the milder forms [76]. The lack of proper characterization becomes even more dramatic, when it comes to molecular genetic testing. Sadly, there is a great discrepancy between different countries, both for financial issues and for prioritization in different public health political strategies [77]. Also, in some cases, even the physicians who are responsible for the patients’ care do not see the importance of genetic testing, as they consider Usher syndrome incurable [78]. The present review has been performed in order to give a complete scenario of the state of the art in the knowledge about this complex and severe condition. While the commonest genes, such as *USH2A* and *MYO7A* are well known and studied, there are very rare causes of this condition that might stay undiagnosed if not specifically investigated [79]. In the next-generation sequencing (NGS) era, still, many laboratories analyze panels of genes related to specific conditions. Although more cost-effective and less time- and work-consuming, this strategy presents the risk of missing rare genes underlying the condition or novel genotype–phenotype correlations emerging from genes related to different conditions.

## 5. Conclusions

In this review, an overview of the present knowledge about Usher syndrome has been given. Particularly, the importance of performing accurate molecular genetic testing for patients and their family members is highlighted, in order to increase the accuracy of genotype–phenotype characterizations. In addition, widening the understanding pathological processes has the aim of building a stronger basis for the appointment of innovative therapeutic strategies.

## Figures and Tables

**Table 1:** Usher 1 Syndrome subtypes, related genes, and percentage of patients genetically characterized [6].

Locus	Gene	Percentage
USH1A	unknown	/
USH1B	*MYO7A*	53–70%
USH1C	*USH1C*	6–15%
USH1D	*CDH23*	10–20%
USH1E	unknown	/
USH1F	*PCDH15*	7–12%
USH1G	*SANS*	<5%
USH1H	unknown	/
USH1J	*CIB2*	rare

**Table 3:** Phenotypic heterogeneity of Usher syndrome. Characteristics symptoms [54].

Usher Syndrome Type	Hearing Loss	Clinically Detectable Vestibular Dysfunction	Retinal Dystrophy
USH I	Congenital, profound	Present	Severe, presenting in the first decade
USH II	Congenital, variable	Not present	Variable severity, usually presenting in the second decade
USH III	Delayed onset, progressive	Variable	Variable
USH IV	Adult onset, variable	Not present	Late onset

## Data Availability

No new data were created or analyzed in this study. Data sharing is not applicable to this article.
